# Transcriptomic analysis by RNA sequencing characterises malignant progression of canine insulinoma from normal tissue to metastatic disease

**DOI:** 10.1038/s41598-020-68507-z

**Published:** 2020-07-14

**Authors:** Y. Capodanno, F. O. Buishand, L. Y. Pang, J. Kirpensteijn, J. A. Mol, R. Elders, D. J. Argyle

**Affiliations:** 1 Laboratory of Fundamental Oncology, National Cancer Research Institute, Tokyo, 103-0045 Japan; 2 Royal (Dick) School of Veterinary Studies and The Roslin Institute, University of Edinburgh, Edinburgh, EH25 9RG UK; 3 Department of Clinical Sciences of Companion Animals, Faculty of Veterinary Medicine, Utrecht University, Utrecht, The Netherlands; 4 London Vet Specialists, 56 Belsize Lane, London, NW35AR UK; 5 Hill’s Pet Nutrition, Topeka, KS USA

**Keywords:** Neuroendocrine cancer, Gene regulatory networks

## Abstract

Insulinomas (INS) are the most common human and canine functioning pancreatic neuroendocrine tumours. The long-term prognosis for malignant INS is poor, because micrometastases are frequently missed during surgery. As human and canine malignant INS share clinical and histopathological features, dogs have been proposed as models for INS research. Using RNA-sequencing, we conducted a pilot study to better understand the underlying molecular mechanisms of canine INS. Normal canine pancreas and lymph node control tissues were compared with primary INS and INS-metastatic lymph nodes, revealing more than 3,000 genes differentially expressed in normal pancreas compared to primary INS. Only 164 genes were differentially expressed between primary INS and INS-metastatic lymph nodes. Hierarchical clustering analysis demonstrated similar genetic profiles in normal pancreas and early clinical stage primary INS, whereas late clinical stage primary INS resembled the genetic profile of INS-metastatic lymph nodes. These findings suggest that markers of malignant behaviour could be identified at the primary site of the disease. Finally, using the REACTOME pathways database, we revealed that an active collagen metabolism, extracellular matrix remodelling, beta-cell differentiation and non-beta-cell trans-differentiation might cause disease progression and hyperinsulinism in INS, identifying major pathways worthy of future research in this currently poorly controlled disease.

## Introduction

Insulinomas (INS) are the most commonly diagnosed functioning pancreatic neuroendocrine tumours in humans and dogs^1,2^. They are insulin-producing tumours that can arise from pancreatic beta-cells^1,2^. Diagnosis and treatment of human and canine INS can be challenging and prognosis highly variable depending on therapeutic decisions^3–5^. Currently, surgical resection of primary localised benign INS is considered the treatment of choice. However, the prognosis after surgery for advanced-stage disease is still poor and adjuvant therapy options are limited^5,6^. Thus, better therapeutic tools are urgently required.

Traditional benign versus malignant classification of these tumours is often difficult at initial diagnosis. Grade and stage are the major determinants of prognosis in humans^4,7^. Similarly, in dogs, TNM stage, increased nuclear size, pleomorphism, Ki67 and DNA content are considered valuable histological features to predict the biological behaviour of INS^6, 8^. Still, grading and staging have been so far insufficient to fully assess the degree of malignancy of human and canine INS. In both humans and dogs, these tumours can have heterogeneous microscopic findings and the presence of metastases, mainly located in the liver, represents so far the only definitive feature that characterises individual tumours as malignant^3,8–11^. Canine INS are considered typically malignant as they develop metastases in 95% of the cases in liver/lymph nodes^9^. Whereas, in humans, they are usually benign as only 5–16% of human INS metastasise^6,12^. If malignant INS could be detected before metastasis, surgery could be curative. Thus, identifying specific INS biomarkers and druggable targets is crucial for earlier diagnoses and efficacious treatments^3–5^.

Previous studies have revealed that many tumours have a unique genetic signature, often with dysregulated gene expression promoting tumour growth and invasiviness^13,14^. Such understanding of INS is limited and there is an unmet need to understand the underlying transcriptomic alterations and molecular changes in order to advance diagnosis and treatment^4^. The lack of knowledge on the genetics of INS might be partly attributed to its low incidence. With only four cases per million population per year, human INS have been previously studied mainly as part of broad studies on pancreatic neuroendocrine tumours (PNETs)^15^. Nonetheless, PNETs are highly heterogeneous tumours thus the specifics of INS oncogenesis are still poorly understood^15^. In dogs, the incidence of INS has not been estimated yet but its rate of malignancy is higher compared to humans^9^.

Comparative oncology aims to study spontaneously occurring tumours in dogs to provide interesting informative models for human cancer research^13,16–18^. Since the completion of the canine genome sequencing^19^, various RNA-sequencing (RNA-seq) studies have identified gene signature characteristics of malignancy in a variety of cancers occurring in dogs such as meningioma^13^, mammary carcinoma^16^, melanoma^20^ and bladder carcinoma^18^, providing additional information about the oncogenesis of human tumours. The close resemblance of canine INS to human malignant INS with regard to clinical signs, histopathology and disease progression, makes canine INS an interesting study model for human malignant INS^21^. For instance, our recent study comparing human and canine INS cell lines identified the Notch pathway as a key regulator of stemness and chemoresistance both in vitro and in vivo, providing a rationale for focused further research on this druggable target for INS in both species^2^.

Given the lack of global gene expression data in canine INS, the aim of this study was to develop integrative computational approaches to identify the mechanisms of tumourigenesis in malignant canine INS. For the first time, comprehensive gene expression analysis of canine INS was performed by RNA-seq to identify key molecular pathways in canine INS. Here, we designed a functional approach to critically analyse the transcriptomic landscape of canine INS and relate malignant molecular features to different clinical stages and pathological features. While the sample size presented herein is not large enough to generate a highly statistically powered analysis of canine INS, the combination of differential gene expression with pathway and network analyses unraveled some of the complex mechanisms in the malignant progression of canine INS that will warrant further investigation in larger studies.

## Materials and methods

### Samples and histopathology

Tumour tissue specimens were obtained from nine dogs with spontaneous INS by partial pancreatectomy at the Faculty of Veterinary Medicine, Utrecht University. Normal pancreas and mesenteric lymph node tissues were obtained for use as controls (Supplementary Table 1). No cases received chemotherapy or radiation prior to surgery. Each tumour tissue was fixed in 10% neutral buffered formalin for 24 h prior to embedding in paraffin. Histopathological examination confirmed a diagnosis of canine INS in all cases based on the most recent adapted canine TNM staging system^6,9^ and WHO grading classification (Supplementary Table 2). Additional information on tissue homogenization is included in Supplementary Materials.

**Table 1:** Top most differentially expressed genes in primary insulinoma vs normal pancreas. In bold genes used for validation with qRT-PCR.

Gene	Gene symbol	Log2FC	*P*-value	FDR	Role
**Pancreatic duodenal homeobox 1**	PDX1	2.13	0.0001	0.003	Beta-cell differentiation
**Paired homeobox 4**	PAX4	6.23	3.32E−09	3.92E−06	
**Insulinoma associated 1**	INSM1	5.021	3.40E−07	5.17E−05	
**NK2 homeobox 2**	NKX2	5.32	3.40E−09	3.92E−09	
**Nestin**	NES	2.53	0.0001	0.003	
Delta/notch-like EGF repeating containing ligand	DNER	4.41	3.05E−07	4.83E−05	Pancreatic ontogeny
**SRY-box 17**	SOX17	2.20	0.009	0.04	
SRY-box 18	SOX18	2.89	0.003	0.02	
**Hes-related family BHLH transcription factor 1**	HEY1	2.46	0.0009	0.01	
Glutathione peroxidase 3	GPX3	4.73	3.91E−08	1.42E−05	Glucose metabolism
Glucokinase	GCK	5.37	3.13E−08	1.23E−05	
Solute carrier 38 family 8	SCL38F8	9.65	9.63E−08	2.35E−08	Insulin secretion
Calcium bynding protein 1	CA1	5.09	1.66E−08	8.82E−06	
Potassium voltage-gated channel subfamily H member 2	KCNH2	5.47	7.40E−09	6.63E−06	
Otoferlin (calcium sensor)	OTOF	5.56	9.07E−08	2.32E−05	
Tetraspanin 1	TSPAN1	5.46	5.95E−08	1.97E−05	Insulin production
Insulin-degrading enzyme	IDE	− 2.29	7.33E−06	0.0003	
Insulin growth factor receptor 2	IGF2	4.33	9.30E−06	0.0004	
**Insulin like 6**	INS	3.21	0.007	0.04	
Insulin receptor	INSR	− 2.62	1.00E−07	2.35E−05	
**Islet amyloyde polyptide**	IAPP	4.37	2.32E−06	0.0001	Amyloid deposition
Chromogranin B	CHGB	4.58	1.33E−07	2.79E−05	Pancreatic neuroendocrine tumours markers
Secretogranin II	SCG2	4.97	1.11E−07	2.49E−05	
Synapsin I	SYN1	5.59	7.87E−09	6.68E−06	
Alpha amylase	AMY1A	− 7.63	8.93E−09	6.88E−06	Pancreatic exocrine marker
Pseudopodium-enriched atypical kinase 1	PEAK1	− 3.09	6.10E−09	5.78E−06	Glucose metabolism
Solute carrier 7 family 1	SLC7A1	− 3.03	2.37E−07	4.13E−05	
Serpin inhibitor peptidase clade I	SERPINA1	3.93	2.08E−07	3.87E−05	Ductal cell markers
Pappalysin 2	PAPPA2	5.40	1.00E−07	2.35E−05	
Glucagon receptor	GCGR	7.63	3.54E−10	1.30E−06	Alpha cell marker
Integrin-alpha 2 (CD49b)	ITA2	− 3.45	2.76E−08	1.17E−05	Cell adhesion

**Table 2:** Top most differentially expressed genes in metastatic lymph nodes vs primary insulinomas. In bold genes used for validation with qRT-PCR.

Gene	Gene symbol	Log2FC	*P*-value	FDR	Role
**Chymotrypsinogen 2**	CTRB2	− 8.72137	0.000178	0.033077	Exocrine markers
**Pancreatic lipase**	PNLIP	− 8.54384	0.000343	0.04279	
**Pancreatic amylase**	AMY2A	− 4.79146	0.000451	0.047458	
**Chymotrypsin-like**	CTRC	− 8.86682	0.000427	0.046186	
**Cytocherathin 19**	KRT19	− 8.38703	0.000115	0.027504	Cell adhesion
Matrix metallopeptidase 23B	MMP23B	− 4.53596	0.000367	0.044113	
Adhesion molecule with Ig like domain 2	AMIGO2	5.298697	8.31E−05	0.02421	
Von Willebrand factor A domain contain 5A	VWF5A	8.34709	3.41E−06	0.026348	
**Serpin peptidase inhibitor, clade I, member 2**	SERPINA1	− 8.809	0.00035	0.043207	Serine peptidase activity
Serine peptidase inhibitor, Kazal type 1	SPINK1	− 8.55149	0.000344	0.04279	
Claudin 10	CLDN10	− 7.19894	0.000195	0.034024	Cell junctions
Claudin 19	CLDN19	− 7.69326	2.40E−05	0.012854	
Gap junction protein beta 1	GAPJB1	5.41801	0.000178	0.033077	
Adhesion G protein-coupled receptor F4	ADGRF4	7.871758	0.000116	0.027504	
C-X-C motif chemokine receptor 5	CXCR5	3.178023	0.000205	0.03415	Inflammation
Chemokine (C-X-C motif) ligand 13	CCL13	4.853102	8.30E−05	0.02421	

### Animals and ethics statement

All patients had been referred to the hospital and were assessed under the supervision of Board-certified specialists in Small Animal Internal Medicine and/or Surgery. Dogs were anaesthetised during surgery under the supervision of Board-certified specialists in Anaesthesia. All diagnostic and surgical protocols were applied for treating patients ensuring their primary welfare according to the Animal Act on Veterinary Practice, as required under Dutch legislation. Written informed consent of each dog owner was obtained for all diagnostic procedures and for the use of anonymised clinical and pathological data for research purposes. Normal pancreas and mesenteric lymph node control tissues were obtained as surplus material according to the University of Edinburgh 3R policy from four dogs that were euthanised for other unrelated research.

### RNA-sequencing and bioinformatic analysis

The RNA-seq reads were generated in Sanger FASTQ™ format using the FastQC software™ (version 0.11.5) (https://www.bioinformatics.babraham.ac.uk/projects/fastqc). FastQC data were trimmed from low-quality reads containing sequencing adapters and more than five unknown bases with Cutadapt™ (version 1.8.3). High-quality paired-end reads from the FASTQ files were mapped to the canine reference genome (CanFam 3.1) resulting in around 95–99% sequences mapped using TopHat2™ (version3.1.84) with default parameters. The output files in the compressed binary version of the Sequence Alignment/Map (BAM) were then assembled and counted using HTSeq™ (version 6.0.1) with mode "union"^22^. Fragments per kilobase of transcript per million mapped reads (FPKM) were imported into R™ (version 3.3.2), and principal component analysis (PCA) was conducted with genes for which the sum of FPKMs of all samples was > 10. Gene counts for each sample were imported into R and differential gene expression was then carried out using edgeR™ (version 3.12.0). Differential expression was assessed for each gene using an exact test analogous to Fisher's exact test but adapted for over-dispersed data. TMM normalization and Tagwise dispersion (individual dispersion for each gene) were used to adjust for abundance differences across samples, and differentially expressed genes (DEGs) were extracted. Genes were considered differentially expressed only at a *P*-value < 0.01 and at False Discovery Rate (FDR) < 0.05. Genes with log2-fold changes of more than 2 or less than − 2 were then selected to ensure that only robust changes were considered. Differentially expressed genes between two groups were annotated using HUGO Gene Nomenclature Committee™ (HGNC) (https://www.genenames.org). Differences in gene expression of pairwise comparisons were organised as follows: (i) normal pancreas against primary INS; (ii) primary INS against INS-metastatic lymph nodes; (iii) normal lymph nodes against INS-metastatic lymph nodes. The normalised gene counts for differentially expressed genes were analysed for hierarchical clustering analysis with the R package “heatmap2”^21,23^.

### Enrichment analysis

Enriched pathways and functional analysis were performed using both Gene set enrichment analysis (GSEA) (https://software.broadinstitute.org/gsea/index.jsp) and Reactome (https://www.reactome.org).

All genes significantly differentially expressed between INS, INS-metastatic lymph nodes and normal tissues, were introduced into GSEA (GSEA version 2.2.2). Gene sets were created from all ensemble genes linked to the database of Canis Familiaris pathways and analysed with Reactome tool to have additional types of annotations to support pathway curation including a variety of biological processes such as signalling, metabolism, transcriptional regulation, apoptosis and synaptic transmission^24^. Gene sets with an FDR < 0.05 were considered as significantly enriched. Additional information on statistical analysis is included in Supplementary Materials.

### Data visualisation

Using Python 3.7.10 (https://www.python.org/) and version 0.19.0 of the Pandas module, the expression data from the DEG, GSEA and Reactome were read into a Pandas data frame. Box plot, smear plot, swarm plot, distribution curve and heatmap were generated in Python using the Matplotlib (version 2.7.1) and Seaborn libraries (version 0.7.1).

### Quantitative real-time PCR

The levels of expression of 13 DEGs were validated using real-time reverse transcription polymerase chain reaction (qRT-PCR). The Platinum Syber Green qPCR Kit (Invitrogen, UK) was used in all qRT-PCR reactions according to the manufacturer’s instructions. Reactions were performed using the Stratagene MX3000P (Agilent, UK). Primer efficiency and dissociation curves were calculated with MXPro software (Agilent, UK), specificity was assessed by agarose gel electrophoresis of the qRT-PCR products as above. Relative gene expression levels were obtained by normalisation to the expression levels of housekeeping genes [Ribosomal protein S5 (*RPS5*) and *GAPDH*)*.* Calculations were made following the Delta Delta Ct Method^25^. The average of the three normal samples was used for relative expression. Additional information on primers and cDNA extraction is included in Supplementary Materials.

## Results

### Quality of the samples and raw data

**Figure 1:**
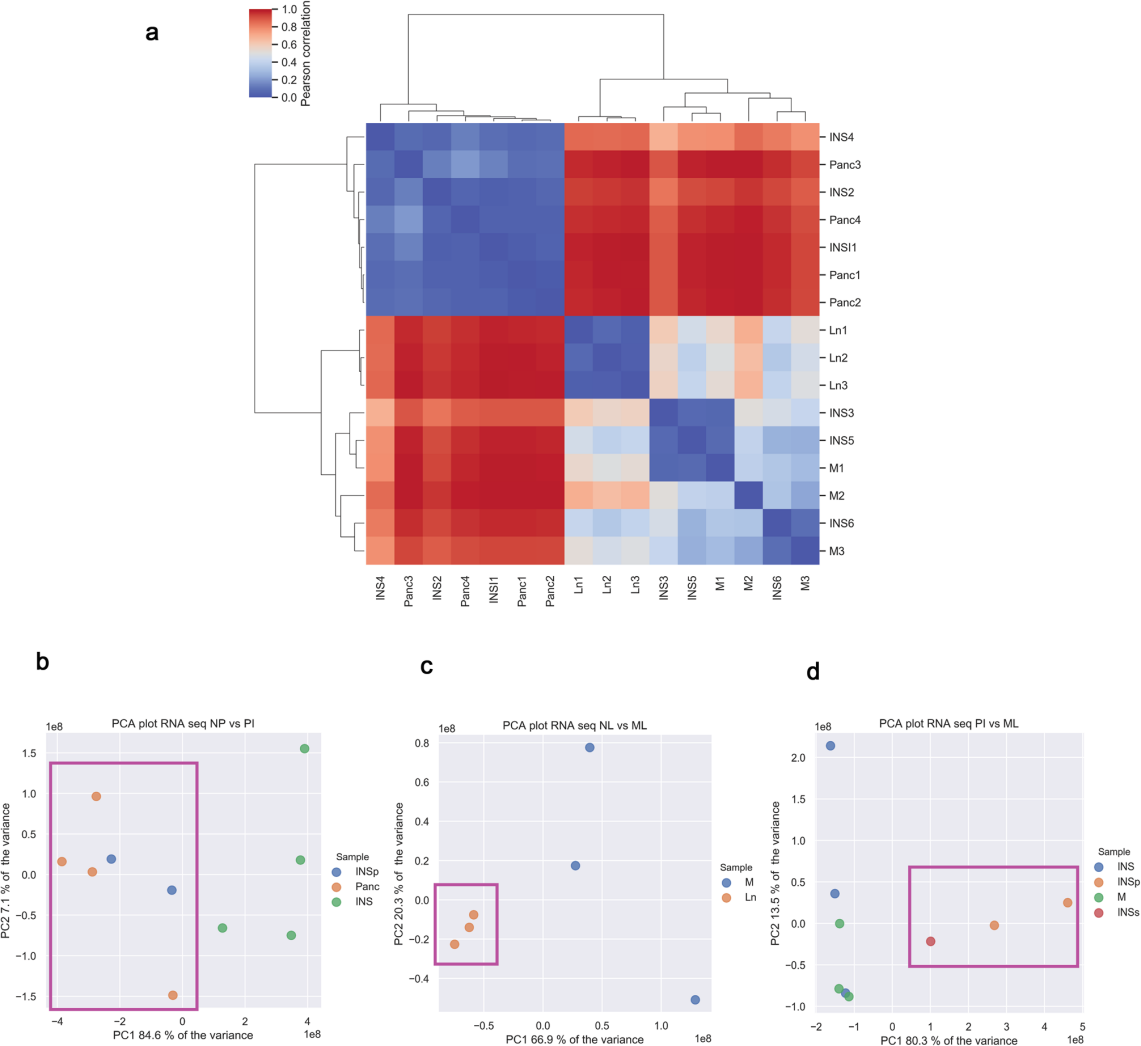
Clustering of normal pancreatic tissues, lymph nodes, primary INS lesions and metastatic lymph nodes. Unsupervised hierarchical clustering based on matrix correlation (**A**) shows clustering of normal pancreatic and lymphatic tissues, primary INS lesions in different TNM stages and metastases. The colour bar indicates the matrix distance (0 = the closest and 1 = the farthest). Multidimensional scaling with PCA plots on two dimensions between normal pancreas and primary INS (**B**), normal lymph nodes and metastatic lymph nodes (**C**), and primary INS and metastatic lymph nodes (**D**) shows controls cluster separately from the tumour samples. Panc = Normal pancreas; INS = Insulinoma TNM stage III–IV; INSp = Insulinoma TNM I stage; INSs = Insulinoma TNM stage II; M = Metastases; Ln = Normal ymph node. Patients’ TNM stage is described in Supplementary Table 1.

For each sample, RNA-seq generated between 50 and 110 million 75 bp paired-end reads and around 95–99% of the reads were mapped to the canine genome. Using unsupervised clustering, two clusters were formed: one including normal pancreatic tissues and primary INS lesions from patients in early clinical stage (TNM stage I and II) and a second cluster included primary INS lesions from patients with late clinical stage (TNM stage III and IV) and metastatic lymph nodes (Fig. 1A). Multidimensional analysis using Principal Component Analysis (PCA) plots showed that the normal pancreatic tissues clustered separately from metastatic and normal lymphatic tissues, whereas primary INS lesions had a more heterogeneous pattern (Fig. 1B–D). MDS analysis showed that normal pancreatic tissues clustered together and INS from patients in early clinical stage clustered together with normal pancreas (Fig. 1B). MDS analysis revealed that normal lymph nodes formed a homogeneous cluster fully separate from INS-metastatic lymph nodes (Fig. 1C). Finally, MDS analysis showed that the distribution of primary INS in early clinical stage clustered separately from INS-metastatic lymph nodes (Fig. 1D). Whereas, primary INS lesions derived from patients in late clinical stage disease had low gene expression variance when compared with the metastatic lymph nodes (Fig. 1D).

### Differential expression analysis of primary INS

**Figure 2:**
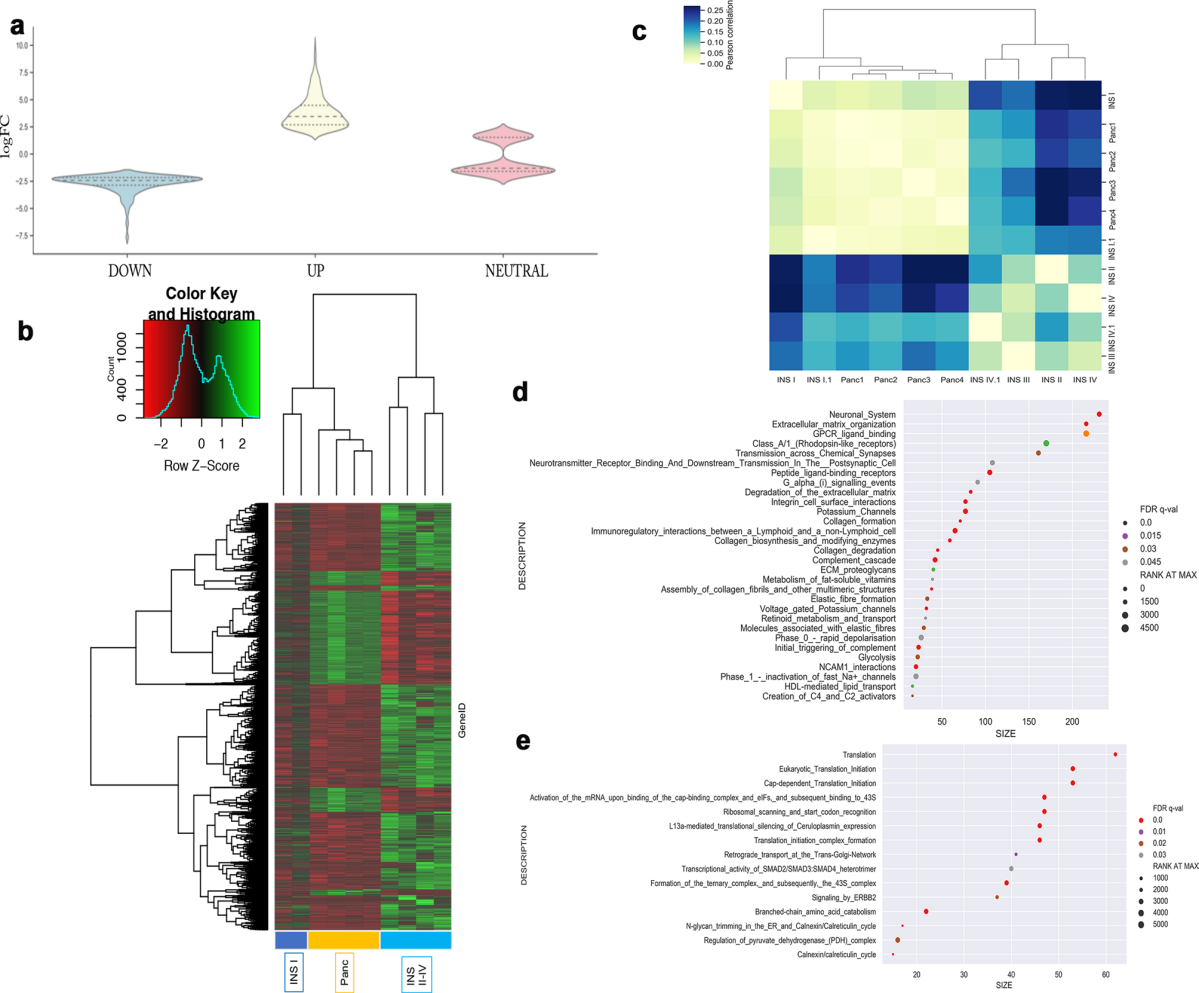
Differential gene expression (DEG) and Reactome pathways analysis between normal pancreatic tissue and primary insulinoma tissue. Violin chart plot (**A**) based on log2FC displays the DEG highlighting the set of altered genes and shows the distribution in the up and down set with log2FC <  − 2 and > 2. Based on the 3,000 differentially expressed genes normal pancreas and primary INS were clustered using heatmap2 function (**B**) and matrix correlation (**C**). Reactome analysis of the enriched function according to Gene set enrichment analysis showed the enriched pathways in the upregulated (**D**) and the downregulated (**E**) set selected on the False discovery rate (FDR < 0.05). Where the size represents the number of genes in the pathway and the Rank at Max shows the presence of these genes between the top up- or downregulated. Patients’ stage is described in Supplementary Table 1.

Paired statistical tests (*P*-value < 0.01; FDR < 0.05) revealed 3,212 genes to be differentially expressed between primary INS and normal pancreas. Violin plot (Fig. 2A), smear plots and volcano plots show the distribution of DEG in the up- or downregulated sections based on their counts and FDR (Supplementary Fig. 1A,B). When only looking at log2-fold changes of more than 2 or less than − 2, 1,900 features remained (1,590 upregulated genes and 310 downregulated genes). Based on the expression levels of DEGs, we performed hierarchical clustering analysis using heatmap functions and revealed distinct clustering of INS tissues in advanced clinical stage (TNM stage II to IV) compared with INS TNM stage I and normal pancreatic tissues respectively (Fig. 2B). Further unsupervised matrix clustering confirmed these findings (Fig. 2C).

When interrogating the 100 most DEG, data revealed many of the upregulated genes were involved in beta-cell differentiation such as Paired homeobox 4 (*PAX4*), NK Homeobox 2 (*NKX2*) and Insulinoma associated 1 (*INSM1*), insulin secretion [tetraspanin1 (*TSPAN1*), glucokinase (*GCK*), insulin (*INS*) and Nestin (*NESTIN*)] and in pancreatic development such as Hes-related family BHLH transcription factor 1 (*HEY1*), pancreatic duodenal homeobox 1 (*PDX1*) and SRY-Homeobox 17 (*SOX17*) (Table 1) (Supplementary Fig. 2). Of interest, well-known pancreatic neuroendocrine markers such as Chromogranin B and Secretogranin II and also alpha and ductal cell-related genes such *GCGR*, *SERPINA1* and *PAPPA2* were also among the top 100 DEG in primary INS (Table 1).

To investigate global trends in the DEG signature, GSEA was performed. Gene sets with significant enrichment included 26 down- and 60 upregulated functions. To further consider the biological significance of these data, they were analysed with the Reactome pathway database which revealed 30 upregulated and 15 downregulated pathways. Most of the upregulated pathways were related to five functional clusters: beta cell fate, insulin secretion/membrane polarization, cell cycling, extracellular matrix organization and collagen remodelling (Fig. 2D). Whereas, about 50% of the downregulated pathways were associated with ribosomes, transcription, and translation of proteins (Fig. 2E).

### Differential expression analysis of metastatic INS

RNA-seq data revealed 6,349 DEG between normal and INS-metastatic lymph nodes (Supplementary Fig. 3A,B). According to hierarchical clustering analysis using heatmap function (Supp Fig. 3C) and matrix correlation (Supplementary Fig. 3D) normal lymph nodes and INS-metastatic lymph nodes formed two separate clusters with different gene expression profiles. These data confirmed that the INS-metastatic lymph nodes had a different gene expression profile compared to the normal lymph nodes.

**Figure 3:**
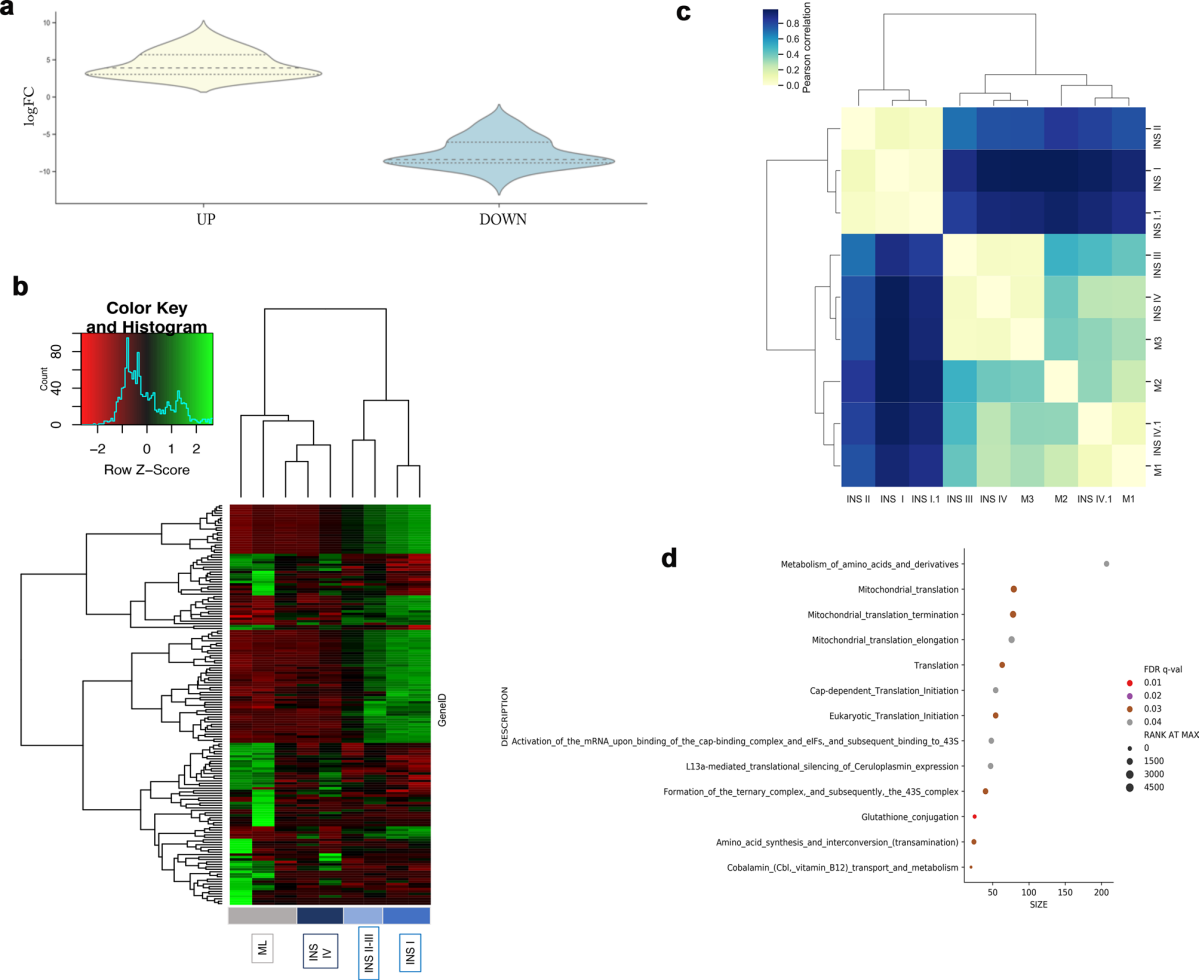
Differential gene expression (DEG) and Reactome pathways analysis between primary insulinoma (INS) tissues and INS-metastatic lymph node tissue. Violin chart plot (**A**) based on log2FC display the DEG highlighting the set of altered genes and the distribution in the up and down set with log2FC <  − 2 and > 2. Based on the 164 differentially expressed genes primary INS and metastatic lymph nodes were clustered using heatmap2 function (**B**) and matrix correlation (**C**). Reactome analysis of the enriched function according to Gene set enrichment analysis shows the enriched pathways in the downregulated (**D**) set selected based on the False discovery rate (FDR < 0.05), where the size represents the number of genes in the pathway and the Rank at Max shows the presence of these genes between the top up- or downregulated. Patients’ stage is described in Supplementary Table 1.

When comparing primary INS tissues and INS-metastatic lymph nodes 164 DEGs were identified with log2-fold changes or more (80 genes upregulated and 84 downregulated). Violin plot (Fig. 3A), smear plots and volcano plots show the distribution of DEG in the up- or down regulated sections based on their counts and FDR (Supplementary Fig. 1C,D). Heatmap analysis showed two separate clusters, one including INS-metastatic lymph nodes and primary lesions derived from patients with TNM disease stage IV and high-grade disease (Grade 2) and the second including primary INS TNM stage I–III samples and low-grade disease (Grade 1) (Fig. 3B). Unsupervised matrix clustering of the coding profiles showed that primary lesions derived from patients with metastatic disease (TNM stage III and IV) clustered together with the metastatic lymph nodes (Fig. 3C).

Relative expression variances showed that acinar-related genes were downregulated in metastatic lesions such as pancreatic lipase (*PNLIP*) and chymotrypsinogen2 (*CTBR2*) together with different transcripts regulating tight junctions such as claudin 10 (*CLDN10*), claudin 19 (*CLDN19*). Whereas, cell adhesion markers previously related to oncogenesis, such as adhesion molecule with Ig like domain 2 (*AMIGO2*) and von Willebrand factor A domain-containing 5 A (*VWA5A*) (Table 2), were upregulated together with small cytokines involved in inflammation, such as C-X-C motif chemokine receptor 5 (*CXCR5*) and chemokine (C-X-C motif) ligand 13 (*CCL13*) (Table 2). The aforementioned genes showed different levels of interactions (Supplementary Fig. 4). Of interest, we revealed that *VWA5A*, *AMIGO2* and *CCL13* were commonly dysregulated in all three pairwise analyses performed in this study (Supplementary Fig. 5 and Supplementary Table 4).

**Figure 4:**
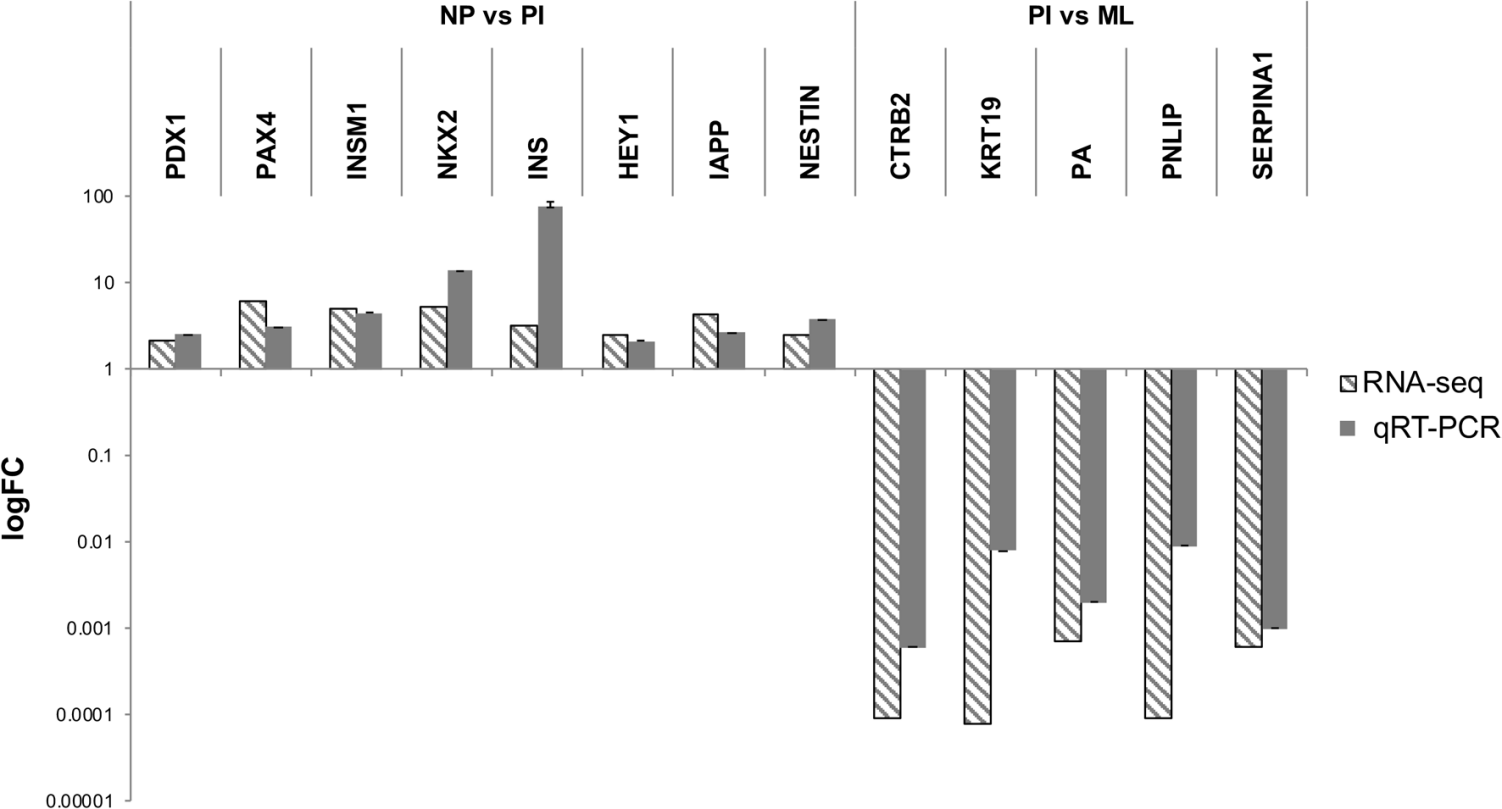
qRT-PCR validation of 13 genes comparing normal pancreatic tissues (NP) and primary insulinoma (PI) and PI and metastatic lymph nodes (ML). The average of three normal samples was used for relative expression (reference delta Ct). logFC = log fold change.

**Figure 5:**
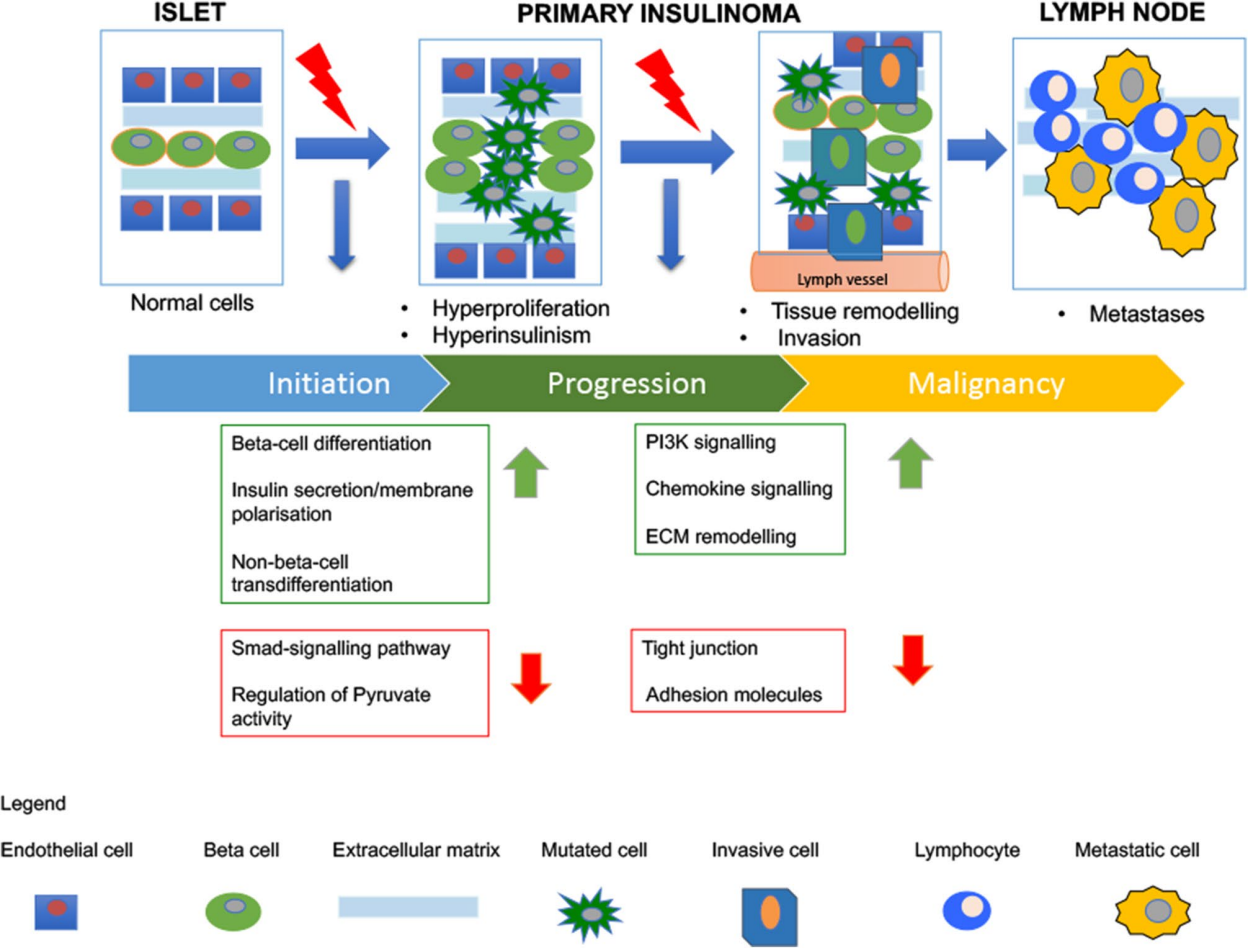
Hypothesised model of canine insulinomas tumour progression. We hypothesise two major changes occur during canine INS oncogenesis towards malignant progression. Early change: upregulation of beta-cell differentiation increases cell proliferation in the normal islets. Then dysregulation of membrane polarisation of cells disrupts the normal insulin homeostasis. Downregulation of Smad-signalling and pyruvate kinase activity further dysregulate the glucose-dependent insulin production. Increased numbers of islet cells and elevated insulin secretion induce a stressful microenvironment that cause trans differentiation of non-beta cells to beta-cells. In this scenario, cell–cell interactions diminish and cells acquire invasive capability. Late change: cell growth in the absence of cell–cell interaction causes loss of their cell adhesion and increase in the extracellular matrix remodelling to facilitate migration towards the lymphatic vessel. An increased cell survival mechanism (PI3K signalling) and increased inflammation (chemokine signalling) push the cells to disrupt the lymphatic vessel and metastasise to the adjacent lymph nodes. These mechanisms together might be responsible for promoting metastatic spread in INS.

Finally, pathway analysis with the GSEA tool through the Reactome pathway database selected on the FDR value, revealed 14 pathways in the downregulated set associated with translation and stress induced response (glutathione conjugation) (Fig. 3D), whereas the PI3K pathway was upregulated.

### qRT-PCR validation of RNA-seq analysis

Expression of thirteen genes was quantified with qRT-PCR to investigate the validity of the results of the RNA-seq transcriptome analysis, using the same RNA template material. Changes in mRNA levels assessed by qRT-PCR were concordant with those observed by RNA-seq analysis. Eight genes were upregulated in the primary INS compared with normal pancreatic tissues including PDX1, INSM1, PAX4, NKX2, SOX17, IAPP, NESTIN and INS. Four genes were significantly downregulated in primary INS compared to metastatic lymph nodes including PNLIP, CTRB2, PA and KRT19. Two endogenous control genes that were expressed in all samples, RSP5 and GADPH were combined to serve as the internal control for each sample. The qRT-PCR results were congruent with the RNA-seq global profiling, supporting the validity of the RNA-seq analyses (Fig. 4).

## Discussion

In this study, we applied an in-depth transcriptome sequencing approach to identify gene expression characteristics of canine INS and to gain new insight into the development of this tumour in dogs.

To the authors’ knowledge, an RNA-seq approach has never before been used to study canine INS, whereas recent studies have examined the genetics of human INS in the much broader context of PNETs^15^. Therefore, not much is known about the genetics of this unique type of tumour. Considering the higher frequency of malignant behaviour in canine INS compared to human INS, our data might offer a model for the further characterisation of the malignant transcriptomic signature of human INS^2^.

Our RNA-seq analysis study detected 3,212 DEGs between normal pancreas and primary INS. Unsupervised clustering of these DEGs showed that normal pancreatic tissues clustered with primary INS lesions from patients bearing early stage INS (TNM stage I), and clustered separately from primary INS lesions from patients bearing advanced stage disease (TNM stage II–IV). When comparing primary INS with INS-metastatic lymph nodes, only 164 DEG were identified, suggesting less transcriptomic variances between these tissues^21^. Unsupervised clustering showed that primary INS from patients with high-grade INS (Grade 2) clustered together with the INS-metastatic lymph nodes. Additionally, unsupervised clustering of normal lymph nodes and INS-metastatic lymph nodes confirmed that the INS-metastatic lymph nodes had a gene signature more similar to the primary INS tissues than to that of normal lymph nodes^21^.

Overall, these data show that early stage/low-grade INS might have a distinct gene expression pattern compared to late stage/high-grade INS. Whereas, late stage/high-grade INS resemble INS-metastatic lymph nodes’ gene profile. Consistent with our data, previous microarray analyses have shown that primary canine INS lesions with high metastatic potential clustered together with their corresponding lymph node and liver metastases, identifying a homogeneous gene signature^9^. Thus, we hypothesise that a specific transcriptomic signature related to a primary mass with high metastatic potential might already belong to primary INS lesions as previously seen in a variety of human cancers including colorectal^26,27^, prostate cancer^28^ and non-functioning PNETs^29^. Given that INS metastases are not easy to detect during surgery^6^, these findings could help to identify those primary INS lesions with a high risk of metastasis based on gene expression.

Amongst the 100 most DEGs between normal pancreatic tissues and INS, we identified beta-cell-specific transcription factors such as PDX1, INSM1, NKX2 and PAX4. Together these genes are key regulators of cell fate in beta-cells and orchestrate a network of genes that govern beta-cell expansion and survival under physiological and pathological conditions^30–32^. Beside beta-cell genes, our data showed that alpha and ductal cell genes such as *GCGR, SERPINA1, PAPPA2* were amongst the upregulated genes in primary INS. These data suggest that the canine INS population is more heterogeneous than previously thought. Previous studies showed that tumour-initiating cells in INS might be of non-islet origin and might derive from ductal and acinar cells^33,34^. For instance, in a zebrafish model, trans-differentiation from alpha cells to beta cells has been identified as a regenerative mechanism of the islets when stress conditions occur independently from glycogen and glucose alteration^35^. Based on these data, we suggest in our model that concurrent overgrowth of beta-cells and trans-differentiation from alpha and ductal cells to beta insulin-producing cells could be involved in tumourigenesis of canine INS^21^ (Fig. 5).

Combined analysis with GSEA and Reactome tools also revealed that upregulated pathways in primary INS compared to normal pancreas were related to three major functional clusters: beta-cell fate, insulin secretion/membrane polarisation and extracellular matrix organisation. Disruptions in beta-cell differentiation have been previously identified in human INS^1,36^, nonetheless the mechanisms causing hyperinsulinism have not yet been elucidated. Mutations of the membrane polarisation in beta-cells could cause the failure of glucose-dependent insulin secretion and these mutations have been related to human diabetes^37^. Our data showed that potassium channel and sodium channel-related functions were amongst the upregulated functions in primary INS. Downregulated pathways in primary INS included the regulation of pyruvate activity and the Smad-signalling pathways. Pyruvate is a key enzyme in glycolysis and in the glucose-related insulin response^38^. Smad-BMP signalling prevented beta-cell differentiation in stem cells in both a zebrafish and a mouse model, and repressed insulin production in islet beta-cell lines and isolated murine islets^39^. These data suggest that the disruption of conductance and membrane polarisation within the cells combined with decrease of the capacity of cells to regulate glucose-related insulin response might be causing loss of normal glucose/insulin homeostasis in INS. These mechanisms together could be the main cause of hyperinsulinism from the early onset of primary INS lesions in dogs^21^ (Fig. 5).

However, different mechanisms seem to be involved in the metastatic progression of malignant canine INS. When comparing primary INS to metastatic lymph nodes we revealed 164 differentially expressed genes^21^. In particular, genes related to acinar exocrine pancreatic enzymes such as *PNLIP* and *CTRB1*, and *PA* together with *KRT19*, a marker of ductal cells and *SERPINA1*, a marker of alpha-cells, were downregulated in the metastatic lymph nodes. These data confirmed the findings of the previous microarray analysis performed on canine INS where these genes were significantly downregulated in the metastatic INS lesions^9^. Two subgroups of extracellular matrix (ECM) proteins were differentially expressed in the metastatic lymph nodes. The first subgroup included two upregulated core matrisome proteins, *AMIGO2* and *VWA5A*. *VWA5A* plays functional roles in cancer progression and in the angiogenic switch of different tumour types including INS^40^. The cell adhesion marker *AMIGO2* has been previously highlighted as a specific neuroendocrine marker^41^ and is involved in liver metastasis^42^. The second subgroup included tight junctions-associated proteins that were downregulated such as *CLAUDIN 10* and *19*. Downregulation of claudins causes loss of cell adhesion, which in cancer is an essential step towards metastatic spread^21, 43^. These results suggested that changes of ECM-cell interaction and collagen-cell interactions might be highly significant during progression of the malignant disease as previously seen in an INS mouse model^40^ but not yet in human INS expression studies^1, 44^.

Consistent with these data, comparisons of the three pairwise genetic analysis revealed that *AMIGO2* and *VWA5A* were upregulated in both primary and metastatic lesions, suggesting that cell adhesion might play a role in canine INS malignant progression. Finally, GSEA and Reactome pathway analysis showed that the remodelling of the ECM was amongst the altered pathways between primary INS and INS-metastatic lymph nodes. Additionally, the PI3K pathway, a well-known oncogenic mechanism in human PNETs^45^, was upregulated in canine INS metastatic lesions. Thus, in our model, we suggest that the dysregulation in ECM remodelling might be an active contributor to driving INS cancer progression, leading to downstream activation of intracellular kinase signalling pathways, such as PI3K. These mechanisms might contribute to generate a fibrotic pro-tumourigenic microenvironment causing collagen modifications and further facilitating the invasion of primary tumour cells into the surrounding tissue (Fig. 5). For instance, recent studies have shown that fibrosis and infiltrative growth pattern are significant risk factors for human PNET recurrence^46^, thus underlining the critical role of the crosstalk between cancer cells and the tumour stroma during PNET tumour progression. Future studies on the whole spectrum of interactions between cancer cells and variable INS tumour stroma components will be needed to fully unravel the role of the mechanisms herein identified in canine INS tumour progression.

In summary, in the current study, we designed a functional approach to critically analyse the transcriptomic landscape of canine INS and relate different malignant molecular subtypes to both clinical staging and pathological grading. Considering the lack of a system to fully characterise INS malignant potential, these findings could significantly help to stratify patients and treat them according to the features of their disease. Additionally, for the first time, we described the changes occurring at the molecular level and the pathways involved from healthy tissues to metastatic disease in canine INS. Our data revealed a number of interesting over- and under-expressed genes, providing rational targets for novel therapeutics and early-stage diagnostics for canine patients. Specifically, we hypothesise that canine INS undergo a complex transformation from the non-neoplastic tissue, that actively promotes collagen metabolism, extracellular matrix remodelling, beta-cell differentiation and disruption of membrane polarisation. These observations provide additional clues to study the mechanisms of hyperinsulinism and metastases often occurring in malignant canine INS. Detailed molecular mechanistic studies would determine the causal relationships of the differentially expressed genes herein identified and INS oncogenesis. Additionally, this model could be easily applied in the future in a large case series to analyse the spatiotemporal axis from disease onset to metastatic disease. Still, these findings elucidate further the process of pathological change from healthy to diseased tissues in canine INS and would support future investigations on druggable targets and explore the translational potential of studying highly malignant subtypes in human and canine INS.

## Supplementary information

Supplementary information

## Data Availability

The sequencing data used in this study have been deposited in the Sequencing Read Archive with accession number PRJNA574196 at the following link (https://www.ncbi.nlm.nih.gov/sra/PRJNA574196). The differential gene expression analysis and functional analysis have been deposited in Figshare repository at the following DOI (10.6084/m9.figshare.c.4686107).
