# Supplementary material for: Transcriptomic analysis by RNA sequencing characterises malignant progression of canine insulinoma from normal tissue to metastatic disease
Source: Sci Rep. 2020 Jul 14;10:11581. doi: 10.1038/s41598-020-68507-z (PMC7360586; doi:10.1038/s41598-020-68507-z)
Supplement: Supplementary file 1 — Supplementary information [file 41598_2020_68507_MOESM1_ESM.pdf]

Transcriptomic analysis by RNA sequencing characterises malignant progression of canine insulinoma from normal tissue to metastatic disease

Capodanno Y[1,2]*, Buishand FO[2,3], Pang LY[2], Kirpensteijn J[3,5], Mol JA[3], Elders R[2,4], Argyle DJ[2]

[1]Laboratory of Fundamental Oncology, National Cancer Research Institute, Tokyo, 103-0045, Japan
[2]Royal (Dick) School of Veterinary Studies and The Roslin Institute, University of Edinburgh, EH25 9RG, United Kingdom
[3]Department of Clinical Sciences of Companion Animals, Faculty of Veterinary Medicine, Utrecht University, The Netherlands
[4]London Vet Specialists, 56 Belsize Lane, London NW35AR
[5]Hill's Pet Nutrition, Topeka, Kansas, USA

*corresponding author: ycapodan@ncc.go.jp

Supplementary Materials and Methods

Tissue homogenisation and RNA extraction

Pancreas tissue was perfused with RNAlater™ (Qiagen, UK) using a 5 mL syringe with a 26-gauge needle in order to obtain a homogeneous distribution of the RNA later throughout the tissue, which was then cut in small pieces and snap-frozen with liquid nitrogen. Mesenteric lymph nodes were collected and immersed in RNA-later and subsequently sectioned and snap-frozen. Portions of INS tissue (5–15 mm diameter) were removed from the central portion of the identifiable mass, placed in cryovials and snap-frozen in liquid nitrogen. All tissues were stored at − 80 °C before RNA extraction. Tissue was homogenised using rotary homogeniser (#SHM2, Stuart, UK) with an additional first step of beta-mercaptoethanol addition to decrease RNases and optimise RNA quality. Total RNA was extracted using the RNeasy Mini Kit™ (Qiagen) To prevent contamination of the samples with genomic DNA, an on-column DNase treatment was performed (Qiagen). RNA concentrations were quantified by spectrophotometry (NanoDrop ND-1000™, Isogen Life Sciences, The Netherlands). RNA integrity was evaluated using an Agilent 2100 Bioanalyzer™ (Agilent Technologies, Santa Clara, CA) according to their RNA integrity number (RIN) values. RIN values above 6.5 were considered acceptable.

cDNA and primers preparation

RNA was transcribed into cDNA using the Omniscript reverse transcription kit (Qiagen) according to the manufacturer's instructions. Where available, previously published primers were checked using NCBI Primerblast (https://www.ncbi.nlm.nih.gov/tools/primer-blast). For primer design, the NCBI nucleotide database was used. For novel primer design, primer melting temperatures (57-63 degrees) were chosen to be similar (forward and reverse), while avoiding primer hairpins, self-dimers and heterodimerisation with the 3'-end of the sequence.

The primer sequences proposed were analysed using the IDT OligoAnalyzer online programme (https://www.idtdna.com/calc/analyzer). Primers were produced by Eurofins Genomics (UK). Primers are listed in Supplementary Table 3.

Statistical analysis

P-values were adjusted for multiple hypotheses corrections using the Benjamini-Hochberg approach, which controls the FDR. Probesets with FDR <0.05 and log2 fold change >2 were considered differentially expressed. All analyses were carried out using R programming language (www.R-project.org).

Supplementary data

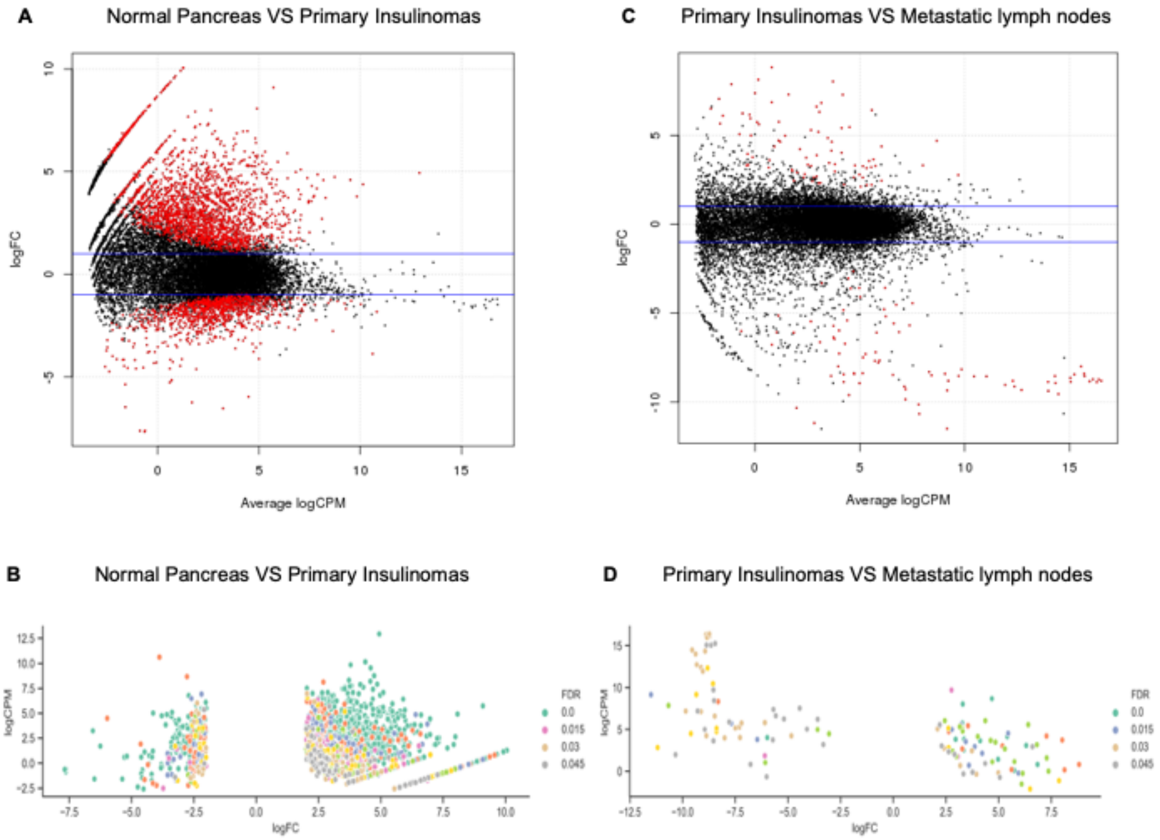

Supplementary Figure 1 Differential gene expression (DEG) analysis between normal mediastinal lymphatic tissue and metastatic lymph nodes. Smear plots and vulcano plots of normal pancreatic tissues versus primary insulinomas (A,B) and primary insulinomas versus metastatic lymph nodes (C,D). Smear plots show gene differential expression comparing log ratio (log2FC) versus abundance (logCPM). Vulcano plots show the distribution of differentially expressed genes with log2FC >2 and <-2 based on their False discovery rate (FDR). FC= fold change; CPM= count per million

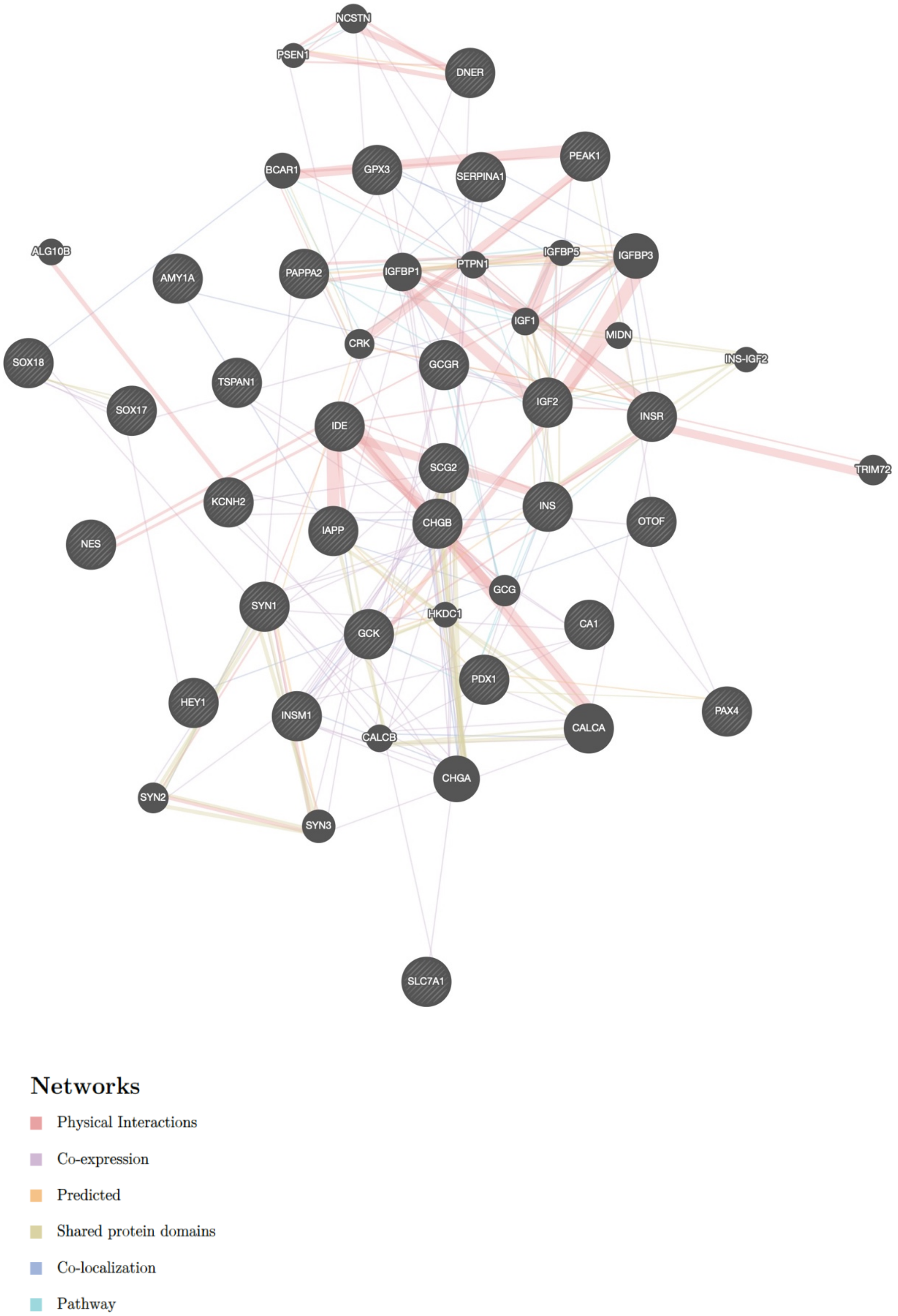

Supplementary Figure 2 Gene networks interactions of the top differentially expressed genes comparing normal pancreas vs primary insulinomas. Graphs made using genemania.com.

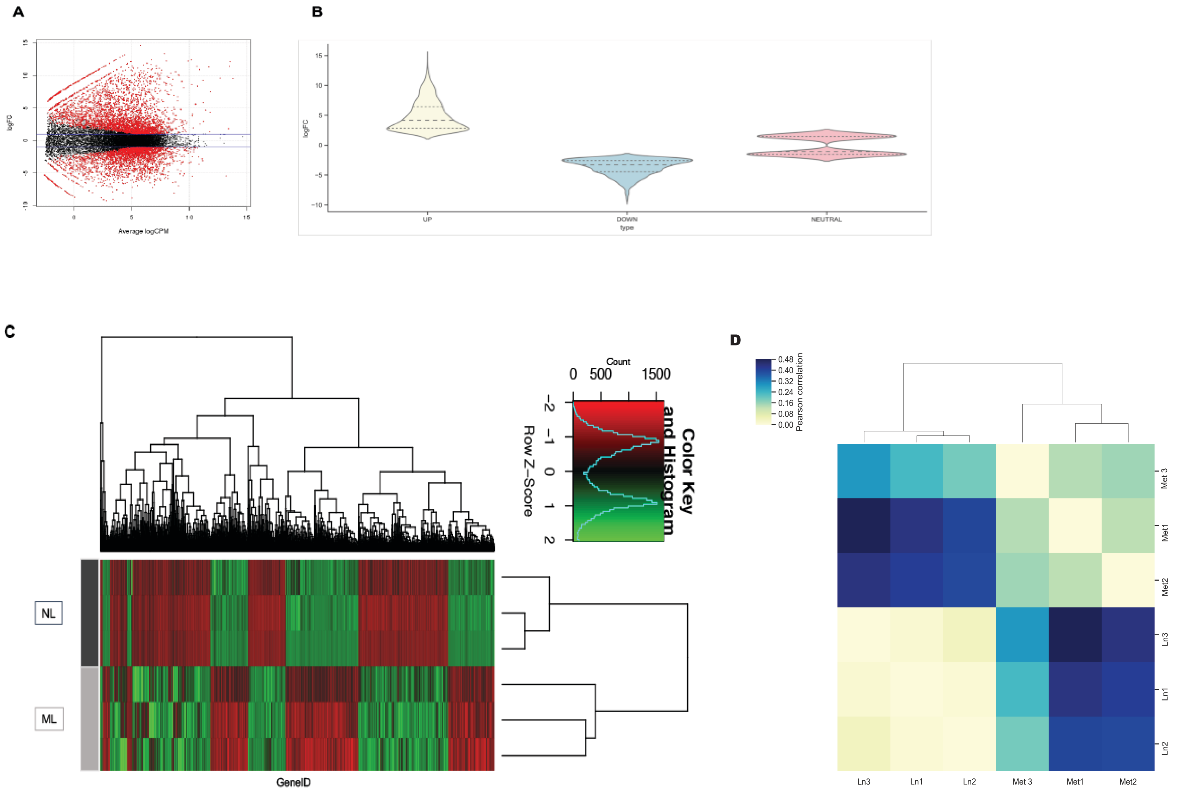

Supplementary Figure 3 Differential gene expression (DEG) analysis between normal mediastinal lymphatic tissue and metastatic lymph nodes. Smear plots (A) using log ratio versus abundance and violin chart plot (B) based on log2FC display the DEG highlighting the set of altered genes. The heatmap analysis of the 6000DEG using heatmap2 (C) and unsupervised matrix clustering (D) shows that the samples separate in two superclusters: one made of normal lymph nodes (Ln) and the second cluster of metastatic lymph nodes (ML).

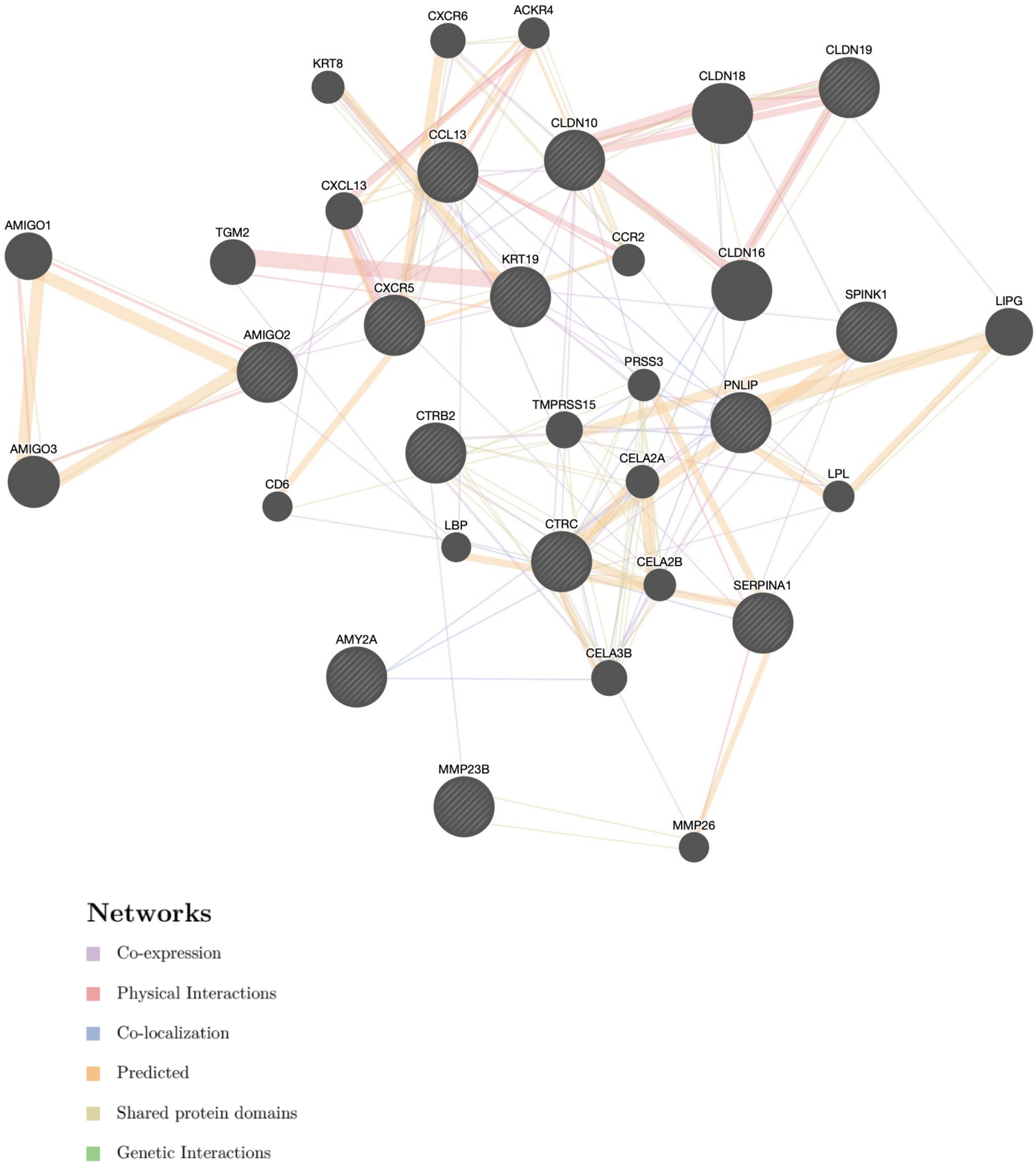

Supplementary Figure 4 Gene networks interactions of the top differentially expressed genes comparing primary insulinomas vs metastatic lymph nodes. Graphs made using genemania.com.

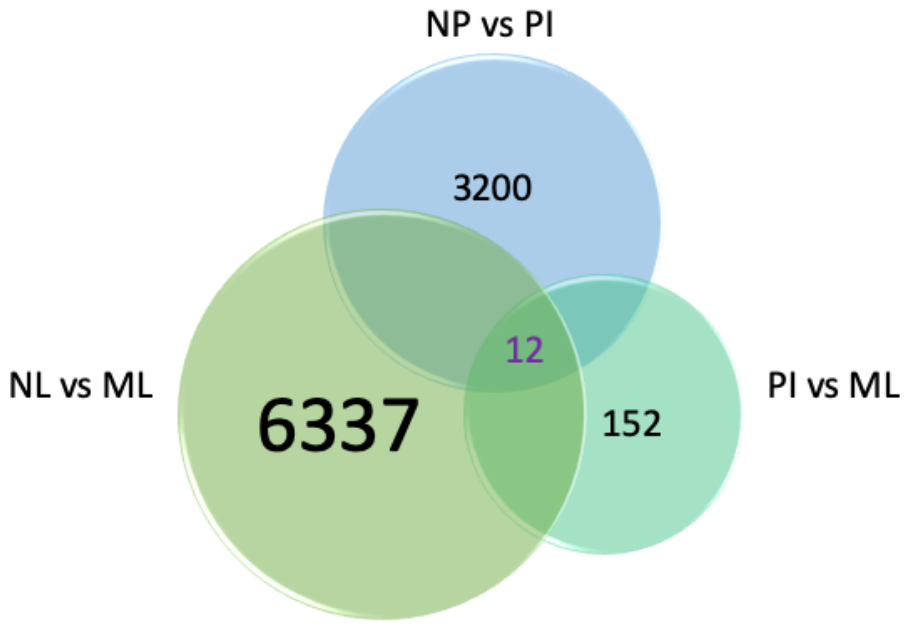

Supplementary Figure 5 Differentially expressed genes overlap between different pairwise comparisons. Venn diagram showing number of overlapping differentially expressed genes between the three pairwise comparisons analysed in this study. NP= Normal pancreas; PI= Primary insulinoma; NL= Normal lymph nodes; ML=Metastatic lymph nodes.

Supplementary Table 1. Clinical features and RNA-quality of control tissues and insulinoma samples used in this study. RIN, RNA integrity numbers; DOD, death of disease (0=alive; 1=dead, LTF=lost to follow-up).

Breed	Age (years)	Sex	Type of sample	RIN value	Concentration (ng/ul)	Abbreviation
**Staffordshire**	10	Female	Control	7.0	490	**Panc4**
**Staffordshire**	2	Male	Control	8.1	185	**Panc1**
				7.5	386	**Ln1**
**West highland**	9	Male	Control	7.7	225	**Panc2**
				9.7	318	**Ln2**
**Pitbull**	2	Male	Control	7.5	240	**Panc3**
				9.2	354	**Ln3**

Breed	Age (years)	Sex	Glucose (mmol/L)	Insulin (mIU/L)	DOD	TNM stage	WHO grade	Type of tissue	RIN value	Concentration (ng/ul)	Abbreviation
**German longhaired pointer**	8	Male	1.0-3.3	unknown	1	I	1	Pancreas	7.9	130	**INS 1 or INS I**
**Irish Soft-coated Wheaten Terrier**	11	Male	3.3	31	0	I	1	Pancreas	6.9	138	**INS 2 or INS I.1**
**Flat coated Retriever**	7	Male	3.0	25	LTF	II	1	Pancreas	6.8	100	**INS 4 or INS II**
**Boxer**	7	Male	2.9	unknown	1	III	1	Pancreas	6.8	151	**INS 3 or INS III**
**Cocker Spaniel**	10	Male	1.6-5.0	unknown	1	IV	2	Pancreas	6.9	200	**INS 5 or INS IV**
**Scottish shepherd**	10	Male	1.5-2.2	unknown	1	IV	2	Pancreas	7.3	100	**INS 6 or INS IV.1**
**Boxer**	8	Male	3.0	46	LTF	IV	2	Lymph node	7.0	150	**M3**
**Malinois**	11	Male	2.2	Unknown	1	IV	2	Lymph node	7.5	400	**M2**
**Crossbreed**	9	Male	3.3	unknown	LTF	IV	2	Lymph node	6.8	190	**M1**

Supplementary Table 2. Adapted TNM staging[6,9] and WHO grading classification for pancreatic neuroendocrine tumours (PNETs) in dogs.

Stage and grade of PNETs in dogs

**T: Primary tumour**			
TX	Primary tumour cannot be assessed		
T0	No evidence of primary tumour		
T1	Tumour limited to the pancreas and size<2cm		
T2	Tumour limited to the pancreas and size 2–4 cm		
**N: regional lymph nodes**			
NX	Regional lymph nodes cannot be assessed		
N0	No regional lymph node metastasis		
N1	Regional lymph node metastasis		
**M: distant metastases**			
MX	Distant metastases cannot be assessed		
M0	No distant metastases		
M1	Distant metastases		
**Stage**			
I	T1	N0	M0
II	T2	N0	M0
III	Any T	N1	M0
IV	Any T	Any N	M1
**Grade**	**Mitotic count (10HPF)**	**Ki-67 index (%)**	
1	< 2	<2	
2	>2.5	>2.5	

Supplementary Table 3. Primers of the thirteen genes used in qRT-PCR

Gene symbol	Primer forward	Primer reverse
**KRT19**	F: GCCCAGCTGAGCGATGTGC	R: TGCTCCAGCCGTGACTTGATGT
**HEY1**	F: ACCTGAAAATGCTGCACACG	R: GCTGGGAGGCGTAGTTGTTA
**SERPINA1**	F: CAACGCCACCGCCTTCTTCATC	R:CCCATTTTGCTCAGGACGCTTTTC
**PA**	F: GGTTCAGATTTCTCCACCC	R: ACTCACAGCATTCCCACAC
**PAX4**	F: GGCACTGGAGAAAGAGTTCC	R: CTTGAGCTTCTCTTGCCGAC
**PDX1**	F: TCCCGTGGATGAAGTCTACC	R: CGTGGCCTCGAGATGTATTT
**INS**	F: CCTTCGTTAACCAGCACCTG	R: CCTTAGGCGTGTAGAAGAAGC
**GADPH**	F:TGTCCCCACCCCCAATGTATC	R: CTCCGATGCCTGCTTCACTACCT
**PNLIP**	F: TGTGTGGACTGGAAGAGTGGC	R: ACAAACTGGGCATCGCTGG
**IAPP**	F:GAGCTGGGAAAGGTGTGAAG	R:ATCCCAAATCTGCTCCTCCT
**INSM1**	F: TGCTAGTGTTCGCTGTGTCC	R: CCAGACTCCAGCAGTTCACA
**RSP19**	F: CCTTCCTCAAAAAGTCTGGG	R: GTTCTCATCGTAGGGAGCAAG
**SOX17**	F: CGACTCTGTTGTGAACCTCC	R: ATAGTTGCAGTAGTACACGGC
**NESTIN**	F:GGTCTCTTTTCTCTTCCGTCCTAA	R: GACCCACTGAGGATGGACAGA
**NKX2**	F: TTCAGTACTCCCTGCACG	R: ATTGTCCGGTGACTCGTC

Supplementary Table 4 List of commonly differentially expressed genes between the three different datasets NP= Normal pancreas; PI= Primary insulinoma; NL= Normal lymph nodes; ML=Metastatic lymph nodes.

Gene	Gene symbol	**NP vs PI DE**	**PI vs ML** DE	**NL vs ML DE**
Adhesion molecule with Ig like domain 2	AMIGO2	Upregulated	Upregulated	Upregulated
Von Willebrand factor A domain contain 5A	VWM5A	Upregulated	Upregulated	Upregulated
Paraoxonase 3	PON3	Upregulated	Upregulated	Upregulated
MHC class I DLA-88 precursor	DLA88	Upregulated	Upregulated	Upregulated
Glutamate decarboxylase 2	GAD2	Upregulated	Upregulated	Upregulated
Tumor necrosis factor receptor superfamily member 11b	TNFRSF11B	Upregulated	Upregulated	Upregulated
Solute carrier family 4, sodium bicarbonate cotransporter, member 8	SCLCA4	Upregulated	Upregulated	Upregulated
Fibrillin 2	FBN2	Upregulated	Upregulated	Upregulated
Angel homolog 1 (Drosophila)	ANGEL1	Upregulated	Upregulated	Upregulated
Chemokine (C-X-C motif) ligand 13	CCL13	Downregulated	Upregulated	Downregulated
Limbic system-associated membrane protein	LSAMP	Upregulated	Upregulated	Upregulated
Pappalysin 2	PAPPA2	Upregulated	Upregulated	Upregulated
